# Massachusetts

**Published:** 1896-05

**Authors:** 


### CONTROL WORK.

*Massachusets.* The Springfield *Republican*, in a recent issue favoring, through its editorial columns, the sustaining of the Tuberculosis Commission in Massachusetts, likened the present experiences of the Board in their use of tuberculin to the organized and determined opposition to and condemnation of vaccination in the early days of its adoption. It voices its opinion in strong support of the work, and quotes from those best able to judge of its real merits, who have been delegated by the Bay State to examine from an impartial point the value of this work. The Commission may well feel proud of the support and earnest recognition of so powerful an ally in all worthy movements as the *Republican* stands for in our land.

Lynn reports licensed milkdealers to the number of 783, a general improvement in the quality of the milk sold, and the arrest and conviction of 28 dealers. Six dairies were stopped from supplying milk owing to their bad sanitary condition. There were 1455 cattle inspected, 369 tested with tuberculin, of which 97 were condemned and killed.

